# Genome-wide identification and expression analysis of the WRKY transcription factor family in flax (*Linum usitatissimum* L.)

**DOI:** 10.1186/s12864-021-07697-w

**Published:** 2021-05-22

**Authors:** Hongmei Yuan, Wendong Guo, Lijuan Zhao, Ying Yu, Si Chen, Lei Tao, Lili Cheng, Qinghua Kang, Xixia Song, Jianzhong Wu, Yubo Yao, Wengong Huang, Ying Wu, Yan Liu, Xue Yang, Guangwen Wu

**Affiliations:** 1 Heilongjiang Academy of Agricultural Sciences, Harbin, 150086 China; 2 Institute of Natural Resources and Ecology, Heilongjiang Academy of Sciences, Harbin, 150040 China; 3 School of Basic Medicine, Guizhou University of Traditional Chinese Medicine, Guiyang, 550025 China; 4 College of Life Science, Northeast Forestry University, Harbin, 150040 China

**Keywords:** Flax, Transcription factor, WRKY, Phylogenetic analysis, Expression patterns

## Abstract

**Background:**

Members of the WRKY protein family, one of the largest transcription factor families in plants, are involved in plant growth and development, signal transduction, senescence, and stress resistance. However, little information is available about WRKY transcription factors in flax (*Linum usitatissimum* L.).

**Results:**

In this study, comprehensive genome-wide characterization of the flax *WRKY* gene family was conducted that led to prediction of 102 *LuWRKY* genes. Based on bioinformatics-based predictions of structural and phylogenetic features of encoded LuWRKY proteins, 95 LuWRKYs were classified into three main groups (Group I, II, and III); Group II LuWRKYs were further assigned to five subgroups (IIa-e), while seven unique LuWRKYs (LuWRKYs 96–102) could not be assigned to any group. Most LuWRKY proteins within a given subgroup shared similar motif compositions, while a high degree of motif composition variability was apparent between subgroups. Using RNA-seq data, expression patterns of the 102 predicted *LuWRKY* genes were also investigated. Expression profiling data demonstrated that most genes associated with cellulose, hemicellulose, or lignin content were predominantly expressed in stems, roots, and less in leaves. However, most genes associated with stress responses were predominantly expressed in leaves and exhibited distinctly higher expression levels in developmental stages 1 and 8 than during other stages.

**Conclusions:**

Ultimately, the present study provides a comprehensive analysis of predicted flax *WRKY* family genes to guide future investigations to reveal functions of LuWRKY proteins during plant growth, development, and stress responses.

**Supplementary Information:**

The online version contains supplementary material available at 10.1186/s12864-021-07697-w.

## Background

Flax (*Linum usitatissimum* L.) is an important industrial crop providing both stem fiber and linseed that are used to produce textiles fiber, edible oil, animal feed, and other industrial products [1]. As of 2011, flax was ranked as the third largest textile fiber crop and the fifth largest oil crop worldwide [2, 3]. Flax is a self-pollinating species with *n* = 15 chromosomes and a genome size of ~ 370 Mb [4, 5]. Bioinformatics analysis of an assembly of a flax whole-genome shotgun library predicted a total of 43,384 protein-coding genes [4]. Although genomic resources in flax are continuously accumulating to accelerate its varietal improvement program [6–11], the genetic basis for the flax fiber development and adaptation to environmental stress has not been fully explored. Therefore, a better understanding of the regulation mechanisms of flax development and stress resistance is critical to make progress and improvements in further flax breeding.

Transcription factors are clue elements in the regulation of signal transduction pathways in living organisms [12]. They often function as central regulators and molecular switches that activate or repress transcription of multiple target genes [13, 14]. The *WRKY* gene family, one of the largest families of transcription factors, has received increasing attention for its members’ roles in plant growth, regulation of defense responses, and stress responses [15–17]. WRKY proteins, which apparently exist exclusively in plants, share a WRKY domain (WD) that is comprised of about 60 amino acid residues [18]. Within the WRKY domain, two conserved sequences are present, a WRKYGQK sequence at the N-terminal end and a C_2_H_2_- or C_2_HC-type zinc-binding motif at the C-terminal end [19–21]. Zinc ions are required for WRKY binding to DNA target sequences, with impairment of binding observed in the presence of metal-chelating agents such as EDTA and 1,10-o-phenanthroline [22, 23]. The specific WRKYs-binding site within a gene promoter is referred to as the W-box. The W-box contains the consensus sequence (C/T)TGAC(T/C) that preferentially binds to all WRKY transcription factors (TFs) except for SPF1 [24]. WRKYs binding specificities for certain promoters may be influenced both by sequences flanking the W-box TGAC core motif and by distinct clustering patterns of functional W-boxes within promoters [24]. WRKY proteins are assigned to three groups (Group I, II, and III) based on number of WRKY domains and zinc finger motif structure [19]. Group I WRKYs contain two WRKY domains and two C–X_4–5_–C–X_22–23_–H–X–H (C_2_H_2_)-type zinc finger motifs. Group II WRKYs contain only one WRKY domain and a C_2_H_2_-type zinc finger motif and proteins of this group have been further subdivided into five subgroups based on phylogenetic relationships (IIa–e). Group III WRKYs contain one WRKY domain and a C–X_7_–C–X_23_–H–X–C (C_2_HC)-type zinc finger motif [19, 25].

Since the first WRKY gene, *SPF1*, was cloned from sweet potato, a large number of WRKY proteins have been identified in a variety of plant species [26–31]. WRKY proteins have been shown to play important roles in growth and development, signal transduction, senescence, and stress resistance [25]. For example, after the *Panax ginseng* gene *PgWRKY6* was cloned and identified by Yang Y et al., it was shown to be upregulated during 2,4-dichlorophenoxyacetic acid (2,4-D)-induced embryogenic callus development; silencing of *PgWRKY6* expression markedly reduced the embryogenic callus induction rate, highlighting the crucial role of this *WRKY* gene in *P. ginseng* hairy root somatic embryogenesis [32]. In *Arabidopsis*, biosynthesis of plant secondary cell walls (SCWs), which are composed mainly of cellulose, xylan, and lignin, has been shown to be regulated by a complex transcriptional network involving WRKYs activities [33, 34]. Specifically, AtWRKY12 was shown to function as a transcriptional repressor, while AtWRKY13 was shown to exert transactivation activity to induce stem lignin biosynthesis through direct *NTS2* promoter binding [35]. Evidence for AtWRKY12 repression of SCW formation was obtained from experimental results showing enhanced SCW formation from pith cells in an *Atwrky12* loss-of-function mutant, while in poplar, PtrWRKY19, a functional ortholog of AtWRKY12, also repressed SCW development from pith cells [36]. Additionally, over-expression of grape Group I VvWRKY2 in tobacco has been shown to alter expression of genes involved in the lignin biosynthetic pathway and cell wall formation [37].

In addition to their cell wall effects, WRKY proteins have been shown to control or modulate plant regulatory networks involving hormonal signaling mediators, including salicylic acid (SA), jasmonic acid (JA), gibberellic acid (GA), abscisic acid (ABA), and ethylene (ET) [38–41]. With regard to plant cell signaling, WRKY transcription factors (TFs), referred to as “jack-of-all-trades” factors, participate in both biotic and abiotic stress responses, with members of all WRKY subfamilies shown to be involved in responses to drought and salt stresses [18]. For example, AtWRKY18, AtWRKY40, and AtWRKY60 Group II subfamily IIa/IIb members negatively regulate transcription of receptor-like kinase CRK5 [41]. Meanwhile, Group I AtWRKY1 TF binds to promoters of *MYB2*, *ABCG40*, *DREB1A*, and *ABI5* to regulate the drought response [42]. In addition, WRKYs can influence salt sensitivity, as Group I AtWRKY8 expression is significantly upregulated in plant roots under salt stress [43]. This observation aligns with results of a study showing that an *AtWRKY8* knockout mutant exhibited greater salt sensitivity (manifesting as growth inhibition) after seed germination as compared to plants with a functional *AtWRKY* gene [44].

Other research has also suggested involvement of WRKYs in microbe-associated molecular pattern-triggered immunity, PAMP-triggered immunity, effector-triggered immunity, and system acquired resistance (SAR) [45]. For example, Group III WRKY PtrWRKY89, a regulator of a poplar SA-dependent defense-signaling pathway, has been implicated in plant pathogen resistance, as overexpression of its SA-inducible gene *PtrWRKY89* led to enhanced expression of pathogen-related (PR) protein genes and improved transgenic poplar pathogen resistance [46]. Meanwhile in *Arabidopsis*, nearly all Group III WRKY members have been shown to respond to diverse biotic stresses, with AtWRKY28 and AtWRKY75 possibly acting via the JA/ET pathway to enhance plant resistance to oxalic acid and fungal infection [47].

The *WRKY* gene family has been suggested to play important and diverse roles in plant growth, development, and stresses tolerance [18]. However, no study to-date has been conducted to identify the *WRKY* genes in the flax genome. Therefore, a thorough investigation of the flax *WRKY* gene family might help to reveal critical molecular mechanisms of flax development and stresses tolerance. In the present study, a comprehensive genome-wide bioinformatics analysis was conducted to predict the flax *WRKY* gene family, yielding 102 *LuWRKY* members. Sequence features, conserved motifs, gene phylogeny, and expression patterns of *LuWRKYs* were also determined. Ultimately, the correlation and co-expression network analyses revealed comprehensive information describing the *WRKY* gene family in flax and provide guidance for future investigations to determine functions of *LuWRKY* genes during flax growth, development, and stress responses.

## Results

### Identification and analysis of *LuWRKY* genes

A total of 107 flax *LuWRKY* genes were predicted using PlantTFDB then their predicted protein sequences were subjected to Pfam and SMART analyses to confirm the presence of WRKY domains. All protein sequences were manually curated and those that did not contain a WRKY domain-like sequence (WRKY signature amino acid sequence with zinc finger motif) were discarded. Five sequences were excluded from further analysis due to their lack of a typical WRKY domain: Lus10001879, Lus10005131, Lus10005132, Lus10007326, and Lus10009969. Finally, 102 sequences were confirmed as flax *WRKY* genes (Table S1). Amino acid number, molecular weight, PI, chromosomal location, conserved motif, and domain pattern for each LuWRKY are listed in Table S1. Lengths of LuWRKY proteins ranged from 82 kD (Lus10022278) to 1199 kD (Lus10012030) amino acids and molecular weights fell between 9.29 kD (Lus10022278) and 132.77 kD (Lus10012030). Predicted PI values ranged from 4.61 to 10.76. Subcellular localization analysis showed that all LuWRKY proteins were localized to the nucleus. Although WRKY domains generally contained a highly conserved sequence (WRKYGQK) together with a zinc finger motif sequence at the N-terminus, numerous variants of the ‘WRKYGQK’ signature sequence were observed, including WRKYGHK, WRKYGKK, WKKYGQK, WRKYDQK, and WRKYHQK, which have altered DNA binding affinity. To facilitate understanding of LuWRKYs functions, already characterized orthologous genes in *Arabidopsis* are also shown in Table S1 based on PlantTFDB.

### Phylogenetic analysis

**Fig. 1:**
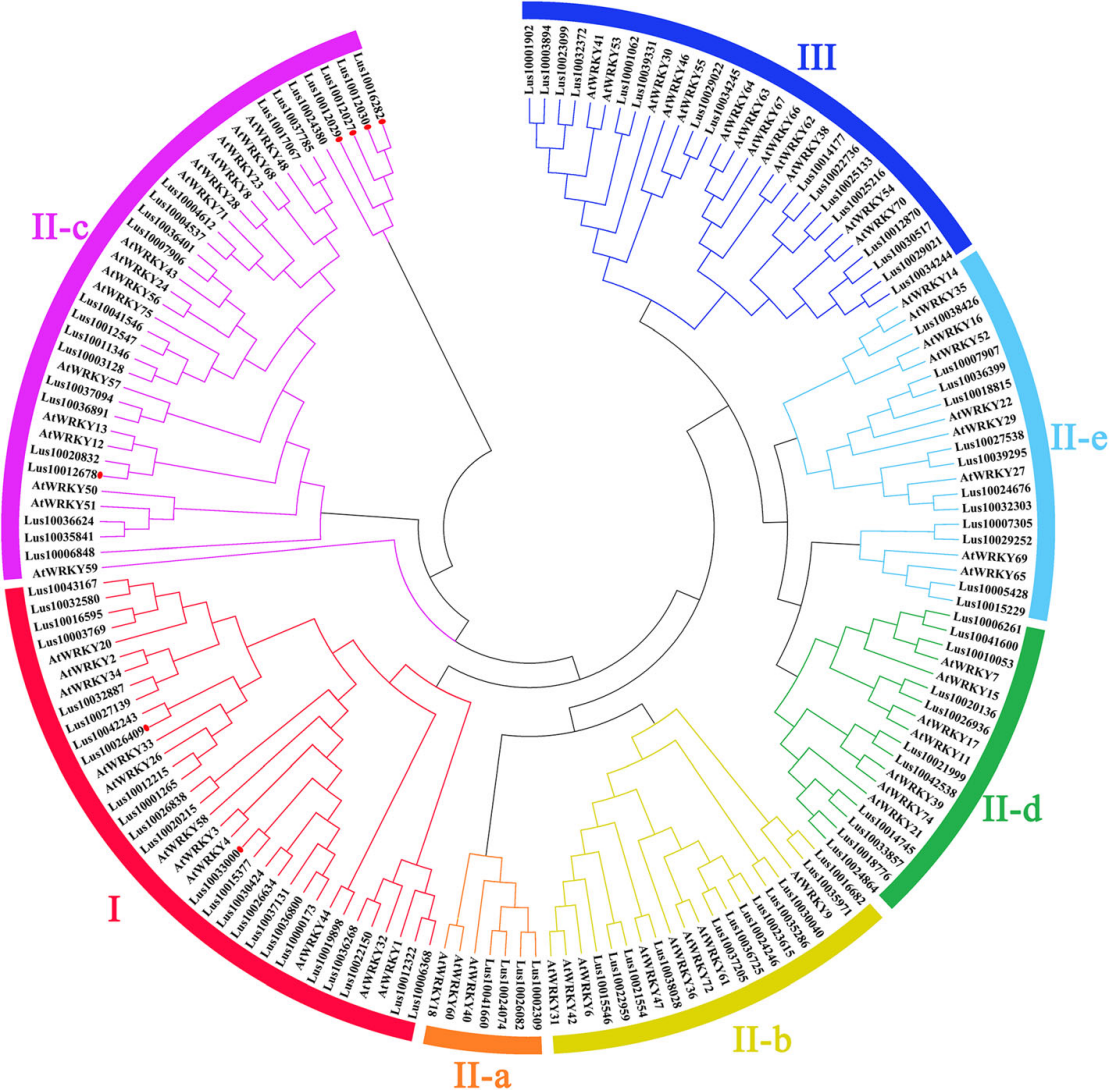
Phylogenetic tree of 101 flax WRKY proteins and 67 *Arabidopsis* WRKY proteins. The phylogenetic tree was constructed using MEGA 5.0 based on the neighbor-joining method, with bootstrap testing performed for 1000 replicates. The seven groups/subgroups are shown in different colors and unclassified proteins are indicated by red circles

To reveal evolutionary relationships of *WRKY* genes in flax and *Arabidopsis,* phylogenetic analyses of 101 LuWRKY and 67 AtWRKY protein sequences were conducted using the neighbor-joining method. Lus10011346 was excluded from the phylogenetic tree because it was too divergent from other sequences to achieve reliable alignment. Diversity was observed with greater prevalence outside rather than within the WD; therefore, full-length WRKY proteins were aligned to maximize the quality of alignments outside the WD and reduce dependency on manual adjustments. Ultimately, 95 LuWRKYs were identified that were assigned to three groups (Group I, II, and III) based on WRKY domain number and type of zinc finger motif (Fig. 1). Group I contained 22 protein sequences that all contained two WRKY domains. Group II and group III protein sequences contained one WRKY domain with various types of zinc finger motifs. The zinc finger motif sequence in Group II was C-X_4–5_-C-X_22–23_-H-X_1_-H (C_2_H_2_), while that found in Group III was C-X_7_-C-X_23–27_-H-T-C (C_2_HC). Of the 57 LuWRKYs assigned to Group II (based on the presence of one WRKY domain and a C_2_H_2_-type zinc finger motif), 4, 11, 19, 11 and 12 LuWRKYs were assigned to Group II subgroups IIa, IIb, IIc, IId and IIe, respectively. Meanwhile, 16 LuWRKYs, each with one WRKY domain and one C_2_HC-type zinc finger motif, were assigned to Group III. Surprisingly, seven LuWRKYs (Lus10012027, Lus10012029, Lus10012030, Lus10012678, Lus10016282, Lus10026409 and Lus10033000) were not assigned to any group, due to their unique structural features that precluded clear assignments into groups/subgroups. For example, Lus10026409 had only one WRKY domain but shared greater sequence homology with Group I members (with two WRKY domains), while Lus10012030 and Lus10016282 had more than two WRKY domains.

### Conserved motif identification

**Fig. 2:**
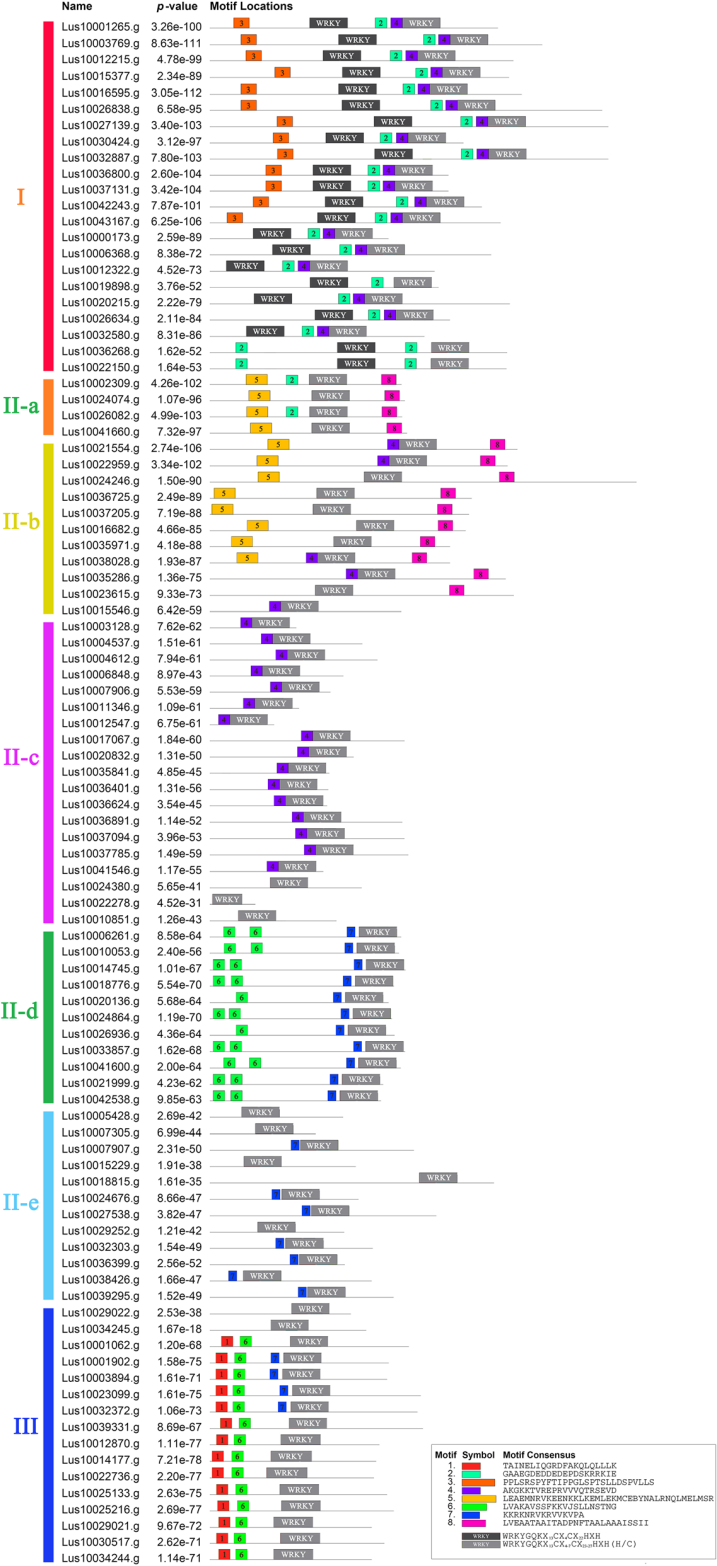
Distributions of conserved motifs in *LuWRKY* genes. Eight putative motifs are indicated in differently colored boxes. N-terminal and C-terminal WRKY domains are indicated in dark and light gray boxes respectively

Conserved motifs of LuWRKY proteins were predicted using the MEME program. A total of eight distinct motifs were identified outside the WRKY domain. As shown in Fig. 2, Group I proteins contained two WRKY domains located at the N-terminus and C-terminus of the protein. Only the C-terminal WRKY domain was present in members of Groups II and III; the C-terminal WRKY domain possessed DNA binding functions. Most LuWRKY proteins within the same subgroup showed similar motif compositions, while high motif composition variability was observed between subgroups. For example, all LuWRKY proteins in Group I possessed motif 2, while all Group IId members contained motifs 6 and 7. Meanwhile, motif 3 and motif 1 were specific to Group I and Group III, respectively, while common motifs 5 and 8 were shared by Groups IIa and IIb and motif 4 was shared by most members of Groups I, IIb, and IIc.

### Expression patterns of *LuWRKY* genes

**Fig. 3:**
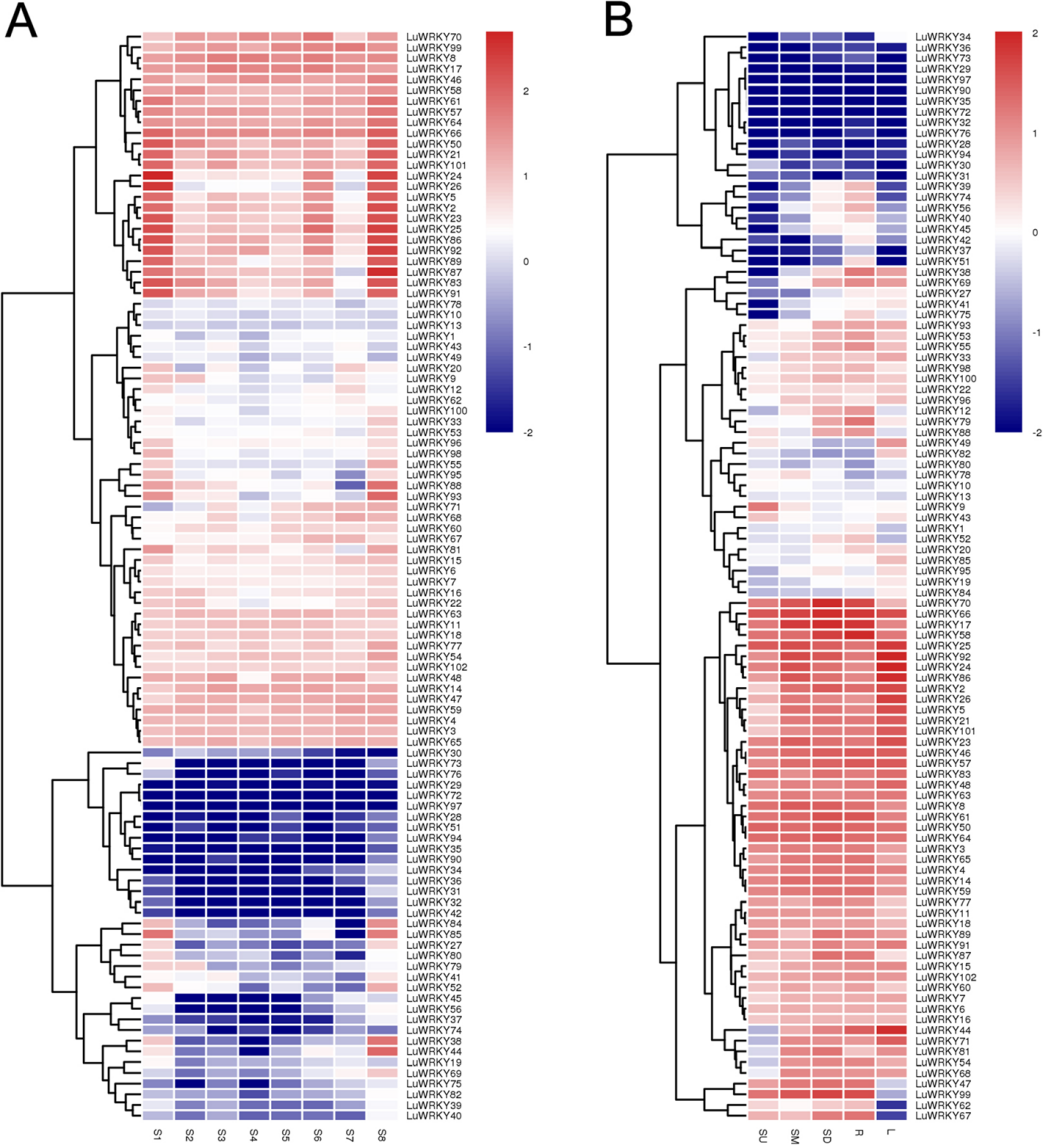
Hierarchical clustering of gene expression levels determined using RNA-seq at different fiber development stages (**a**) and in different tissues (**b**). FPKM values of *LuWRKYs* were transformed by log10. S1, seedling stage; S2, fir like stage; S3, early fast growing stage; S4, fast growing stage; S5, bud stage; S6, flowering stage; S7, green stage; S8, maturity stage. Upper, middle, and lower third zones of stem, root, and leaf at late fast growing stage are designated SU, SM, SD, R, and L, respectively

The data that support the findings of this study have been deposited in the CNSA (https://db.cngb.org/cnsa/) of CNGBdb with accession number CNP0001606. Using RNA-seq data, expression patterns of 102 LuWRKYs were determined and FPKM values of genes encoding these LuWRKYs are shown in Table S2. Among the 102 LuWRKY genes, 14 showed very low levels of accumulated transcripts across all samples (FPKM < 1). These genes may be pseudogenes or they possibly may vary in spatial and temporal expression patterns. Heatmaps for LuWRKY genes showing FPKM values converted to log10 values were constructed using Heml software (Fig. 3).

Next, expression profile data were divided into two parts, with one part related to different fiber development stages (Fig. 3a) and the other part related to relative expression level in different organs (Fig. 3b). As shown in Fig. 3a, 11 of the 102 genes (10.78%) were highly expressed (FPKM > 10) at all developmental stages in stems. In addition, many genes exhibited their highest expression levels at early or late stages of fiber development, including 22 genes (21.57%) at stage 1 and 57 (55.88%) at stage 8; 89 genes (87.25%) were expressed in all three organs (stem, root, and leaf) (Fig. 3b), while 29 genes showed predominant expression in only one tissue, including 3 (2.94%) in stem, 13 (12.75%) in root, and 13 (12.75%) in leaf. Meanwhile, 17 genes were differentially expressed in stem, with expression levels of 14 genes observed to proportionally increase with stem position (i.e., bottom > middle > top) and expression of three genes exhibiting the opposite pattern (i.e., top > middle > bottom).

### Validation of RNA-seq data by quantitative RT-PCR (qRT-PCR)

**Fig. 4:**
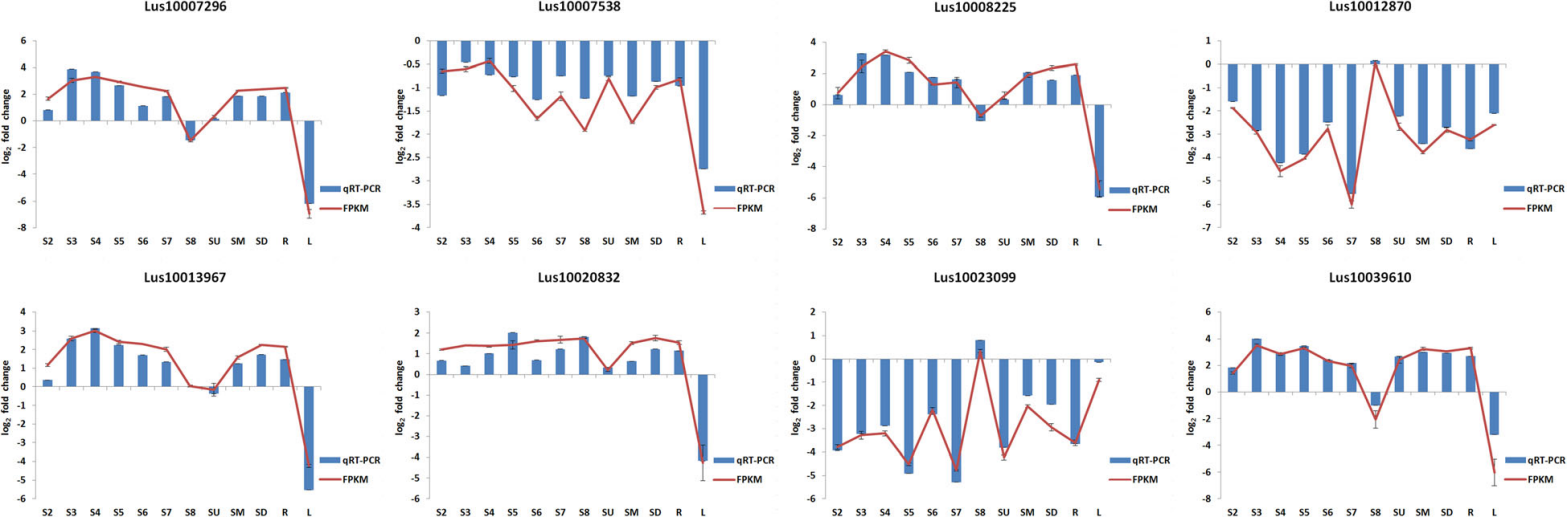
Validation of RNA-seq data by qRT-PCR. The red line represents the value of FPKM in the DGE profile and the blue histogram represents the expression level of eight genes detected by qRT-PCR

To further verify the accuracy of flax digital gene expression (DGE) profiles, the expression levels of eight randomly selected genes were analyzed by qRT-PCR, including *LuCesA8* (Lus10007296), *LuCesA3* (Lus10007538), *LuCesA4* (Lus10008225), *LuWRKY83* (Lus10012870), *LuNAC10* (Lus10013967), *LuWRKY47* (Lus10020832), *LuWRKY86* (Lus10023099) and *LuMyb46* (Lus10039610). The results showed that expression levels of the eight genes determined by qRT-PCR agreed with the results of sequencing analysis and the RNA-seq data were reliable (Fig. 4).

### Correlation analyses

**Fig. 5:**
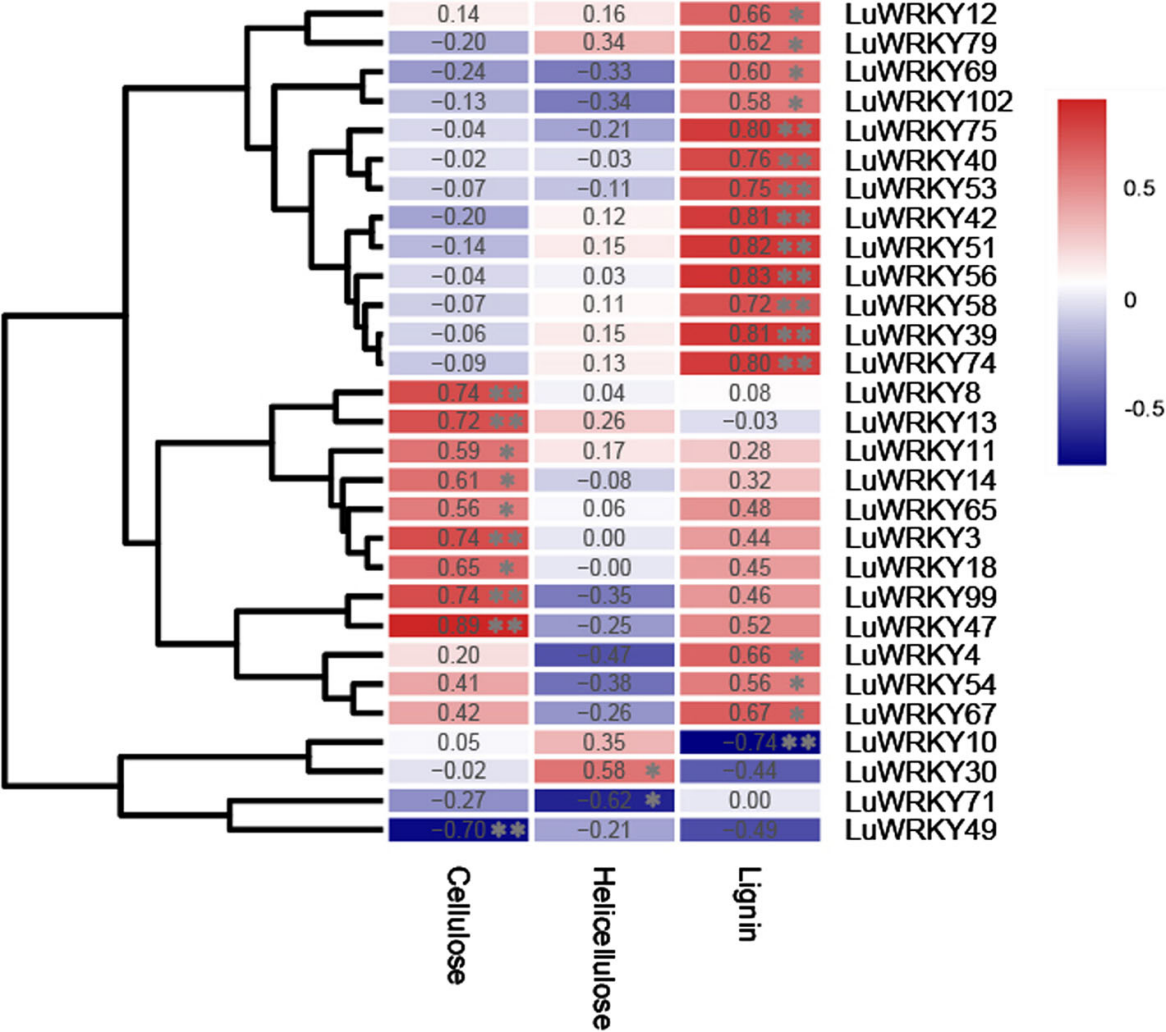
Correlation analyses between *LuWRKY* gene expression and cellulose, hemicellulose, and lignin contents. Pearson correlation coefficients were shown in the box. The level of significance was set to *p* < 0.05. * Correlation is significant at the 0.05 level (2-tailed). ** Correlation is significant at the 0.01 level (2-tailed)

After plant cellulose, hemicellulose, and lignin contents were determined at different developmental stages and in different tissues (Table S3), the correlations between the expression levels of *LuWRKY* genes and the contents of cellulose, hemicellulosic and lignin were analyzed (Fig. 5). Of the total 102 *LuWRKY* genes, expression levels of nine genes showed significantly positive correlations with cellulose content, while only *LuWRKY49* (Lus10024380) was negatively correlated with cellulose content (*p* < 0.05). *LuWRKY30* (Lus10022959) and *LuWRKY71* (Lus10015229) were found to be positively and negatively correlated with hemicellulose content (*p* < 0.05), respectively. Meanwhile, expression levels of sixteen genes showed significant positive correlations with lignin content, and only *LuWRKY10* (Lus10020215) negatively correlated with lignin content (*p* < 0.05). Importantly, these results suggested that correlation analysis was useful for identifying genes that potentially exerted key regulatory effects on cellulose, hemicellulose, and lignin synthesis in flax.

### Co-expression network analysis

**Fig. 6:**
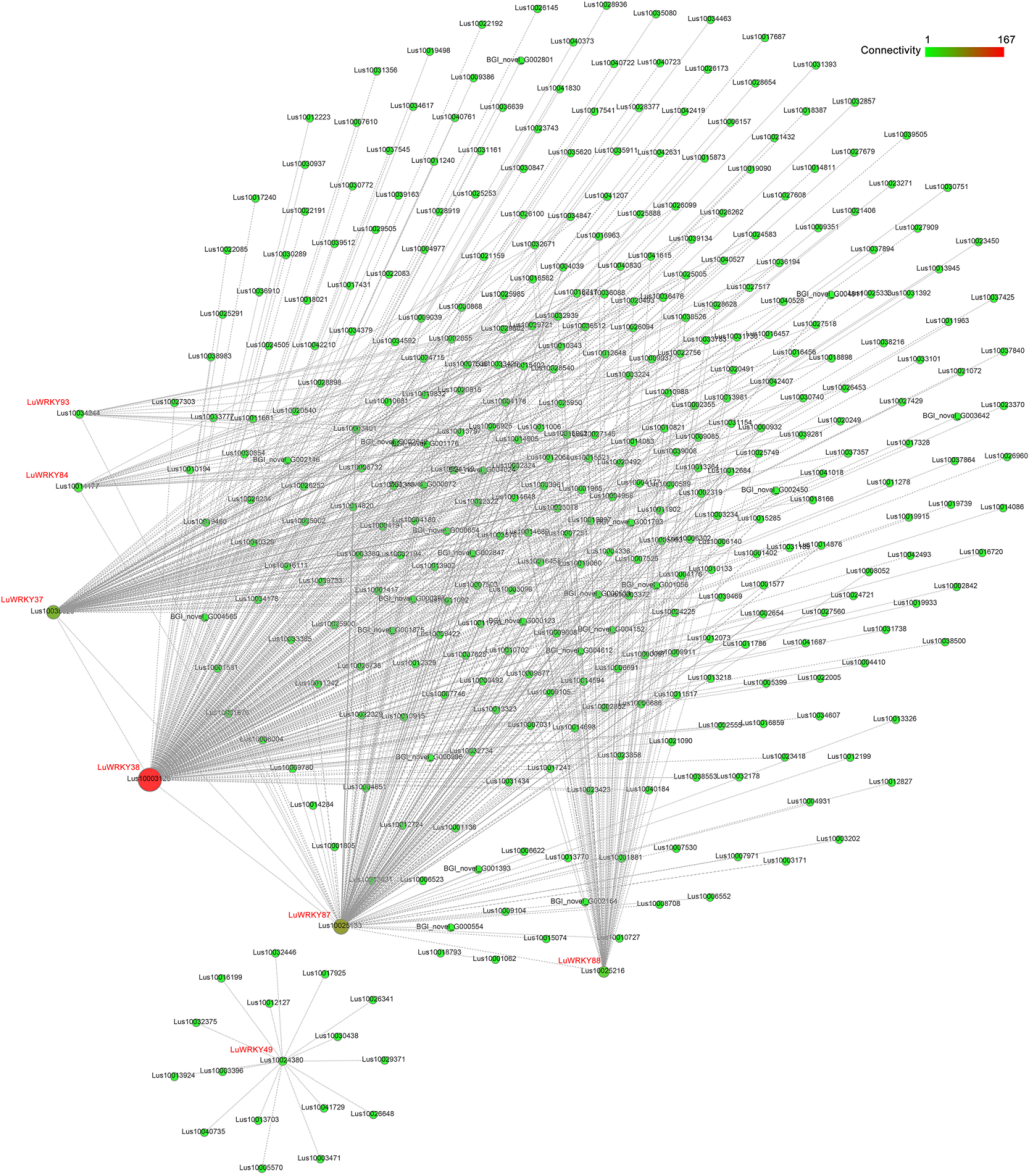
Co-expression genes network of *LuWRKY* genes. The colors of the circle represent the different connectivities and ranges from green (genes with low connectivities) to red (genes with high connectivities)

**Fig. 7:**
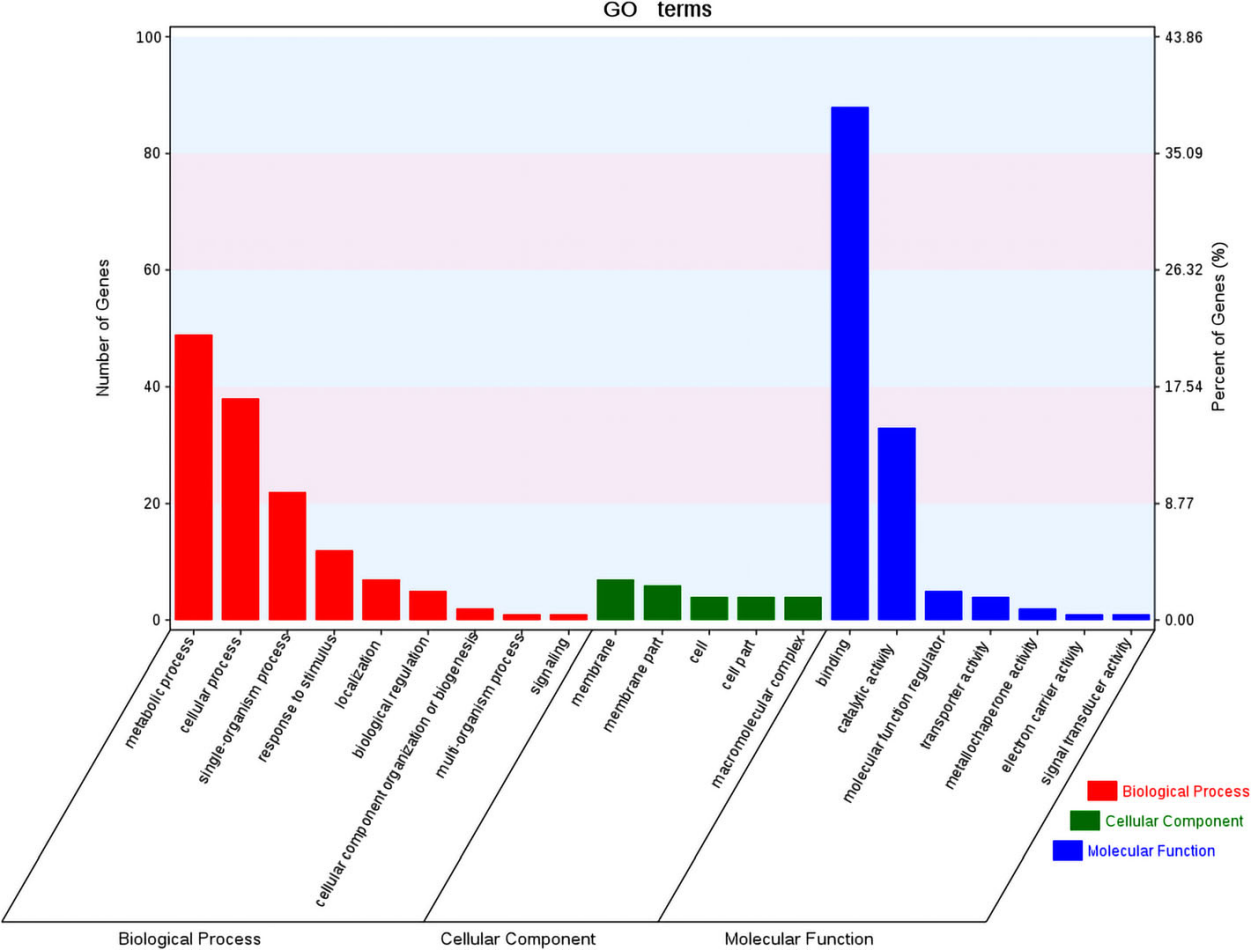
Go enrichment analysis of 228 co-expression genes with *LuWRKTs*. The X-axis represents number of genes, and Y-axis represents different GO terms

A total of 42,886 genes detected in expression profiling data were subjected to weighted gene co-expression network analysis to reveal genes co-expressed with *LuWRKY*s (based on screening for proteins with scores above 0.5). After the co-expression network was constructed and visualized using Cytoscape (Fig. 6), seven *LuWRKY*s genes, including *LuWRKY38* (Lus10003128), *LuWRKY84* (Lus10014177), *LuWRKY49* (Lus10024380), *LuWRKY87* (Lus10025133), *LuWRKY88* (Lus10025216), *LuWRKY93* (Lus10034244), and *LuWRKY37* (Lus10038028), were identified as hub genes with high co-expression correlations with 361 other genes. Table S4 lists co-expressed genes with correlation coefficients. Of 361 identified co-expressed genes, 228 were annotated using the GO database (Fig. 7). The GO term “binding” (GO: 0005488) best described the greatest number of genes (88), while the GO term “metabolic process” (GO: 0008152) best described 49 genes, and the GO term “cellular process” (GO:0009987) best described 38 genes.

## Discussion

The WRKY TF family ranks as the seventh largest TF family in plants, after basic helix-loop-helix (bHLH), myeloblastosis-related (MYB), ethylene responsive factor (ERF), NAC (NAM, no apical meristem, ATAF1/2, and CUC2, cup-shaped cotyledon), basic leucine zipper (bZIP), and C_2_H_2_ TF families [45]. Although *WRKY* genes appear to exist in some diplomonads, social amoebae and other amoebozoa, and members of fungal class incertae sedis, *WRKY* genes are absent in other non-plant species [48]. The first cDNA encoding a WRKY protein, SPF1, was cloned from sweet potato (*Ipomoea batatas*) [26]. The WRKY family arose during evolution through tandem and segmental gene duplication. To date, 14,549 *WRKY* genes from 166 plant species have been deposited in PlantTFDB [47], including 72 WRKYs in *Arabidopsis*, 116 in cotton, 103 in rice, and 104 in poplar [45]. Genome information of flax (*L. usitatissimum*) revealed that whole-genome duplication (WGD) had occurred in the lineage of *L. usitatissimum* between 5 and 9 Mya [4] and subsequently gave rise to the 102 flax genes identified as *WRKY* genes in the present study.

Based on phylogenetic analyses and WRKY domain structures, 95 of the 102 LuWRKYs were assigned to seven groups (Groups I, IIa, IIb, IIc, IId, IIe, and III). Twenty-two WRKY proteins possessing two WRKY domains (at the N terminus and C terminus) were assigned to Group I, while those possessing a single C-terminal WRKY domain were assigned to Group II or III. Importantly, the two WRKY domains of Group I members have distinct functions; the C-terminal domain plays a major role in binding to the W-box, while the function of the N-terminal WRKY domain remained unclear and might influence promoter binding specificity and affinity [23, 49]. Notably, only the C-terminal WRKY domain is responsible for sequence-specific binding to DNA, as AtWRKY1 recognition of the W-box appears to mainly depend on the presence of the C-terminal WRKY domain, while the presence of the N-terminal WRKY domain only slightly influenced the protein–DNA interaction [23, 50]. Many variants of ‘WRKYGQK’ signature-sequence are present in LuWRKYs, including WRKYGHK, WRKYGKK, WKKYGQK, WRKYDQK, and WRKYHQK [51].WRKY domain show high affinity binding to a DNA sequence, termed the W-box sequence (C/T)TGAC(C/T), which is found in the promoter region of many genes [52].

In addition to the W box, a recent study indicates that the WRKY domain can also bind to SURE, a sugar responsive cis element, as a transcription activator [53]. In group-IId WRKYs, a plant zinc-cluster domain (PF10533) was present upstream of the WDs. The LuWRKY proteins in group III are typified mainly by having the less common CX_7_CX_23–27_HXC zinc binding motif [19]. It has been reported that substitutions of the WRKYGQK residues in the WRKY domain decreased the DNA-binding affinity, and any mutations of the conserved cysteine and histidine of the zinc-binding motif abolished the protein–DNA interaction [51].

The *WRKY* genes are believed to have originated approximately 1.5 to 2 billion years ago in eukaryotes prior to the divergence of plant phyla; this phylogeny clearly aligns with results of a recent report outlining the evolution of *WRKY* genes in flowering plants [48]. Based on our phylogenetic motif analyses here, we propose four major WRKY transcription factor lineages in flax: Groups I + IIc, Groups IIa + IIb, Groups IId + IIe, and Group III. These lineages align with a previous hypothesis asserting that a proto-WRKY ancestral gene with a single WRKY domain underwent domain duplication to produce Group I WRKY genes; thus, an ancestral Group I WRKY would have given rise to all *WRKY* genes. Subsequent loss of the N-terminal WRKY domain led to Group IIc genes. In the present study, along with clear division of most LuWRKY proteins, some exceptions were also present. For example, the WRKY domain structures of LuWRKY96 (Lus10012027), LuWRKY97 (Lus10012029), LusLuWRKY98 (Lus10012030), LuWRKY99 (Lus10012678) and LuWRKY102 (Lus10033000) were intermediate type between group I and group IIc. The LuWRKY101 (Lus10026409) proteins belong to group-IIc based on WD structure, but it was clustered with the group-I members in phylogeny. The results were consistent with the previous reports and indicated that the group-IIc was more evolutionarily close to group-I than other groups [54]. The presence of this PR intron in Group IId, IIe and III WRKY genes supports the hypothesis that these groups evolved from the group I C-terminal domain [48, 55]. In addition, Group III genes appear to share a common motif 7 with the Group IId + IIe, which indicated that the three groups were adjacent in evolutionary relationship. The recent work on the evolution of the WRKY gene family, proposed Group IIe genes predate Group IId genes [24] and Group III genes evolved earlier than groups IIa and IIb [56]. Group IIa genes are the group with the smallest number of members in flax. However, the lack of clustering of members of flax Group IIa and *Arabidopsis* Group IIa implies that diversification occurred after the divergence of monocots and dicots. In addition, all of the main groups of WRKY genes that are present in flowering plants are present in *Selaginella moellendorffii* except for Group IIa genes which were therefore the last to evolve and might appear to have arisen from Group IIb genes [48].

Numerous studies have shown that *WRKY* genes play crucial roles in diverse physiological and developmental processes [19, 41, 57]. Specifically, *AtWRKY2* and *AtWRKY34* are redundantly involved in pollen formation, pollen tube elongation, seed germination, and early growth after germination [58]. *Lus10027139* and *Lus10032887*, homologues of *AtWRKY2*, are predominately expressed in plant stems and show a high level of expression during fiber development in flax. This implies that these homologues might be potential regulators of fiber formation in addition to their role in regulating pollen formation and seed germination. Almost all plant cells possess primary cell walls; however, some specialized cells, such as fiber cells, form thickened secondary cell walls. The deposition of SCWs provides mechanical strength, enhanced water-conducting capabilities [59, 60], and a defense structure to prevent pathogen entry into cells. In flax, bast fibers are produced that have very thick SCWs that contain high amounts of cellulose (> 70%) with low lignin content (2–7%). Indeed, the synthesis of SCWs in numerous plant species involves activity of WRKYs. For instance, SCW biosynthesis in potatoes is regulated via a complex transcriptional network [34], with StWRKY1 exerting direct control over secondary cell wall thickening through its action on the promoters of hydroxycinnamic acid amide (HCAA) biosynthetic genes, encoding 4-coumarate-CoA ligase (4-CL) and tyramine hydroxycinnamoyl transferase (THT). In grape plants, *WRKY2* plays a role in regulating lignification, while tobacco plants over-expressing *VvWRKY2* exhibit altered expression of genes involved in lignin biosynthesis and cell wall formation [37]. In *Arabidopsis*, AtWRKY13 has been reported to bind the *AtNST2* promoter and regulate *AtNST2* gene expression during SCW synthesis associated with sclerenchyma cell development [35, 61]. In the present study, correlation analysis indicated that expression levels of 29 *LuWRKY*s could be significantly correlated with cellulose, hemicellulose, or lignin content. These genes were mainly categorized into Groups I + IIc or Groups IId + IIe, with expression profiling data showing that most of them were expressed predominantly in stem. Both *Lus10020832* and *Lus10012678*, homologues of *AtWRKY13,* showed significantly positive correlations with cellulose content, implying their putative roles in SCW formation. *Lus10024380*, a homologue of *AtWRKY49,* exhibited significantly negative correlations with cellulose content, which may function as a negative regulator and repress SCW biosynthesis in flax*.* Moreover, *Lus10006368*, *Lus10016595*, *Lus10037094*, *Lus10033857*, and *Lus10042538*, homologues of *AtWRKY1*, *20*, *57*, *21*, and *74,* respectively, displayed similar expression patterns and up-regulated expression during flax fiber development. Their expression levels were usually lowest in the top part of the stem, gradually increased in the middle, and were highest at the bottom part of the stem. The results strongly suggest that these genes likely play key roles during fiber development [62].

In addition to SCW synthesis*, WRKY* genes have been also shown to play important roles in responses to various abiotic stresses, including drought, salt, heat, and osmotic stresses [45]. For example, *AtWRKY15* modulates plant growth and mediates salt/osmotic stress responses in *Arabidopsis* [63]. CRK5, a receptor-like protein kinase, is involved in abscisic acid (ABA) signaling and drought tolerance. *AtWRKY18/40/60* negatively regulates the transcription of CRK5 in *Arabidopsis thaliana*. Meanwhile, *AtWRKY25/26/33* genes have demonstrated to participate in heat-induced signal transduction [64]. Corresponding to these characterized *Arabidopsis WRKYs*, transcripts of the flax orthologues of *AtWRKY15* (Lus10006261, Lus10041600), *AtWRKY33* (Lus10001265, Lus10012215, Lus10042243, Lus10026409), and *AtWRKY40* (Lus10002309, Lus10024074, Lus10026082) showed significant induction under saline-alkaline stress [65]. In addition, *AtWRKY46/54/70* genes belong to Group III and encode important signaling components that regulate BR-regulated growth and osmotic stress [66]. Among the orthologous genes of *AtWRKY46/54/70* in flax, *Lus10012870*, *Lus10025133*, *Lus10025216*, and *Lus10030517*, identified in response to BR [67], *Lus10012870* and *Lus10030517* were involved in flax osmotic resistance [68]. Expression profile data indicate that the genes, including *Lus10001265*, *Lus10002309*, *Lus10012215*, *Lus10012870*, *Lus10024074*, *Lus10026082*, *Lus10026409*, and *Lus10043167*, exhibited very similar expression patterns in the current work. They showed distinctly higher expression levels at stages 1 and 8 than the other stages and were predominantly expressed in leaves. The majority of these genes are classified into Groups I, IIa, and III. However, these genes have yet to be functionally characterized in flax successfully. We speculate that the genes might be promising candidate regulators involved in stress tolerance in flax.

WRKYs have also been shown to function as a hub to integrate signaling of multiple plant defensive phytohormones (JA, SA, ABA, GA, ET) during regulation of disease resistance and biotic stress responses [69]. Expression of AtWRKY7, a negative regulator of plant defense signaling during infection with bacterial pathogen *Pseudomonas syringae*, is induced by SA and *P. syringae* [70], while AtWRKY57 expression, which also negatively regulates plant defense signaling to infection, increases susceptibility of plants to *Botrytis cinerea* [71]. Conversely, AtWRKY4 enhances plant resistance to both necrotrophic and biotrophic pathogens, while upregulated OsWRKY71 levels induced by SA, methyl jasmonate (MeJA), or pathogen infection leads to enhanced resistance to *Xanthomonas oryzae*, as observed for an OsWRKY71 overexpression mutant [72]. With regard to plant antifungal defenses, AtWRKY28 and AtWRKY75 may enhance plant resistance to fungal infection through the JA/ET pathway [73], while LuWRKY36, a homolog of AtWRKY33, appears to promote secoisolariciresinol biosynthesis in response to *Fusarium oxysporum* elicitors [74].

Lignin is both developmentally deposited and pathogen-induced in the secondary thickened cell wall [75]. As a defensive chemical barrier, lignin plays important roles in preventing pathogen invasion. In fact, defense-induced lignification is a conserved basal defense mechanism employed by a wide range of plant species. In cotton (*Gossypium hirsutum*), quantitative analysis of resistance to wilt fungus *Verticillium dahliae* revealed an association between increased lignification in stems upon infection and resistance against wilt [76]. Meanwhile, transgenic tobacco plants constitutively overexpressing lignification-enhancing phenylalanine ammonia lyase (PAL) genes exhibited greater resistance to pathogens *Cercospora nicotianae* and *Phytophthora parasitica* cv. Nicotianae [77, 78], while RNAi-mediated suppression of expression of the gene encoding cinnamyl alcohol dehydrogenase (CAD) (normally expressed during normal vascular cell wall lignification) increased flax susceptibility to vascular fungus *Fusarium oxysporum* [79]. Fundamentally, little is known regarding the distinction between vascular cell wall lignification and defense-induced lignification in plants and their precise regulatory mechanisms. Nevertheless, in this study, the expressions of *Lus10026634*, *Lus10004537*, *Lus10004612*, *Lus10036891*, *Lus10037094*, *Lus10041546*, and *Lus10010053* belonging to Group I + IIC were correlated with lignin biosynthesis, while their homologues, such as *AtWRKY4*, *7*, *57*, *71*, and *75*, in *Arabidopsis* are known to play important roles in plant response to various biotic stresses. It remains unclear if these LuWRKYs also participate in the regulation of defense-induced lignification in flax. Therefore, further studies are needed to determine the function of the LuWRKYs in immune regulation.

## Conclusion

In this study, we conducted a genome-wide search for flax *WRKY* gene family members that led to identification of a total of 102 *WRKY* genes. Subsequent bioinformatics-based analyses of WRKY proteins revealed LuWRKYs amino acid numbers, molecular weights, predicted isoelectric point (PI) values, chromosomal locations, domain patterns, and conserved motifs. LuWRKYs were phylogenetically classified into three groups (Groups I, II, III), with Group II further divisible into five subgroups, for a total of seven LuWRKYs subgroups. Using RNA-seq data, expression patterns of *LuWRKY*s were determined at different developmental stages in diverse tissues. Notably, expression of 10, 2, and 17 genes were found to be significantly correlated with the cellulose, hemicellulose, and lignin contents, respectively. Moreover, many LuWRKYs were also shown to play important roles in responses to various biotic and abiotic stresses. The results of this study present comprehensive information describing the *WRKY* gene family in flax and provide useful clues to guide future investigations to determine functions of *LuWRKY* genes during flax growth, development, and stress responses.

## Methods

### Plant materials

The fiber flax variety ‘Diana’ was used in this study. Plants were grown in the experimental field of the Industrial Crops Institute of Heilongjiang Academy of Agricultural Sciences (Harbin, P.R. China) under natural conditions. They were planted in a 4-m^2^ (2,0 m × 2,0 m) plot with 2000 plants per m^2^ with a raw spacing of 20 cm. The soil of the experimental plot was chernozem (pH of 6.8). Hand weeding was used and there are no irrigation or fertilization treatments.

For analysis of differential gene expression profiles and determination of fiber chemical composition, plant samples were collected at different stages. The middle third of the stem was collected at eight different stages of flax fiber development: seedling stage (4th pair of true leaves unfolded), fir-like stage (stem, 10% of final length), early fast growing stage (stem, 30% of final length), fast growing stage (stem, 50% of final length), bud stage (visible flower buds), flowering stage (50% of flower open), green stage (seeds green and undeveloped) and maturity stage (plants are developed for harvesting of fiber type). In addition, the upper (9–15 cm from the shoot apex), middle (33–39 cm from the shoot apex), and lower (57–63 cm from the shoot apex) sections of the stems, roots (main root and fine root), and leaves (middle section) were also collected during the late fast growing stage (stem, 80% of final length, length of plant 72 cm).

For DGE analysis, thirteen samples were collected, and three biological replicates were produced for each sample. The samples used for chemical composition determination were prepared in triplicate, and 5 individual plants were pooled as one replicate. After samples were collected, they were immediately frozen in liquid nitrogen then stored at − 80 °C.

### RNA extraction

Each plant tissue sample (kept frozen in liquid nitrogen) was ground into a fine powder using mortar and pestle. Plant total RNA was extracted using the cetyl trimethylammonium bromide (CTAB) method. For each sample, 4 μg of total RNA was digested in a 25-μl total volume with DNase I (Promega, Madison, WI, USA) to remove genomic DNA contamination. RNA quality was checked via 1.0% agarose gel electrophoresis followed by RNA visualization and quantification using an Agilent 2100 Bioanalyzer (Agilent Technologies, Santa Clara, CA, USA).

### DGE library preparation and sequencing

Sequencing data was filtered using SOAPnuke (v1.5.2) [41] by (1) removing reads containing sequencing adapter; (2) removing reads whose low-quality base rate (base quality less than or equal to 5) was > 20%; (3) removing reads whose unknown base (‘N’ base) frequency was > 5%. After filtering, clean reads were stored in FASTQ format then were mapped to the reference genome using HISAT2 (v2.0.4) [80]. Bowtie2 (v2.2.5) [81] was applied to align clean reads to the reference coding gene set then expression levels of genes were calculated using RSEM (v1.2.12) [82].

### RNA-sequencing (RNA-seq) data analysis

High-throughput sequencing analysis software (HTSeq-v0.5.3) was used to enumerate the number of fragments mapped to each gene. Based on gene lengths and fragment counts mapped per gene, fragments per kilobase per million mapped fragments (FPKM) values were calculated for each gene in conjunction with sequencing depth and gene length ranges for fragment counts. Ultimately, FPKM values were used to estimate gene expression levels [83].

### Identification of *LuWRKY* genes in flax

Protein sequences and DNA-binding domains of WRKY proteins were obtained from the Plant Transcription Factor Database (PlantTFDB) at (http://planttfdb.cbi.pku.edu.cn/). All candidate genes were further examined by confirming they contained WRKY core sequences using PFAM (http://pfam.xfam.org) and SMART (http://smart.embl-heidelberg.de/) online tools. Basic information about these genes, including amino acid numbers, molecular weights, predicted isoelectric points (PIs), conserved motifs, and domain patterns, were acquired through PlantTFDB. The chromosomal location was obtained through the Phytozome12 website (https://phytozome.jgi.doe.gov/). Subcellular localization was predicted using Cell-PLoc 2.0 website tools (http://www.csbio.sjtu.edu.cn/bioinf/Cell-PLoc-2/).

### Analysis of phylogenetic relationship and conserved motifs

Full-length amino acid sequences of WRKYs derived from *Arabidopsis* were obtained using online phytozome12. Multiple alignments of 101 LuWRKYs and 67 AtWRKYs protein sequences were performed via ClustalW (version 1.83) using default parameters. A phylogenetic tree was constructed using the neighbor-joining method of MEGA 5.0 with 1000 bootstrap replicates. Conserved motifs of LuWRKYs were identified via the MEME program (version 5.1.1, http://meme-suite.org/tools/meme) using the following parameters: any number of repetitions, maximum of 10 motifs, and an optimum motif width of 6 to 60 amino acid residues.

### Expression pattern analysis of *LuWRKY* genes

RNA-seq data expressed as FPKM was downloaded to study expression patterns of *LuWRKY* genes. To render the data suitable for cluster displays, absolute FPKM values were divided by the mean of all values then ratios were transformed into log10 values. HemI 1.0 software was used to generate the heatmap then heatmap analysis was performed using OmicShare tools, a free online platform for data analysis (http://www.omicshare.com/tools).

### Quantitative RT-PCR analysis

cDNA synthesis was performed using 1 μg of DNase I-treated RNA using the PrimeScript RT Reagent Kit (TaKaRa, Japan) according to the manufacturer’s protocol. qRT-PCR was performed to determine transcript levels with quantification performed using an Opticon machine (Biorad, Hercules, CA, USA) after amplification using a real-time PCR Mix Kit with SYBR Green fluorescent dye (TOKOBO). To normalize variance among samples, the stably expressed *GAPDH*, *EF1A* and *ETIF5A* genes were used as internal controls [84]. The middle third of the stem at seedling stage was used as the sample normalizer. The relative expression levels were calculated from the threshold cycle according to the 2^-ΔΔCt^ method [85] and the experiments were carried out in triplicate to ensure reproducibility of each sample. Gene-specific primers were designed using Primer 5.0 software and primer sequences are shown in Table S5.

### Correlation analyses

Cellulose, hemicellulose, and lignin contents were detected in plant samples using the contents detection kits (QIYI, Shanghai) via UV spectrophotometry. The procedures were performed following the instructions of the kits. Cellulose content was measured by the anthrone method, and hemicellulose content was detected using the 3,5-Dinitrosalicylic Acid (DNS) method. The acetylbromide method was employed to determine the lignin content.

### Co-expression network analysis

Normalization and processing of expression profile data were performed using the R software package. The normalized dataset was modularized using a weighted gene co-expression network analysis (WGCNA) algorithm. Genes co-expressed with *LuWRKY*s were screened based on threshold value > 0.5 then filtered genes were used to construct the correlation network. The network was visualized using Cytoscape version 3.6.1 (www.cytoscape.org).

## Supplementary Information

**Additional file 1: Table S1.** The character of the WRKY proteins identified in flax.

**Additional file 2: Table S2.** The FPKM values of the *WRKY* genes in flax.

**Additional file 3: Table S3.** The contents of cellulose, hemicellulose, and lignin at different developmental stages and in different tissues.

**Additional file 4: Table S4.** The co-expressed genes with the *LuWRKY* genes.

**Additional file 5 Table S5.** Primers used for qRT-PCR analysis.

## Data Availability

All data generated or analysed during this study are included in this published article and its supplementary information files.

## Abbreviations

ABA: Abscisic acid
bHLH: Basic helix-loop-helix
bZIP: Basic leucine zipper
CAD: Cinnamyl alcohol dehydrogenase
CTAB: Cetyl trimethylammonium bromide
DGE: Digital gene expression
DNS: 3,5-Dinitrosalicylic Acid
ERF: Ethylene responsive factor
ET: Ethylene
FRDL4: Ferric reductase defective-like 4
GA: Gibberellin
HCAA: Hydroxycinnamic acid amide
JA: Jasmonic acid
MeJA: Methyl jasmonate
Myb: Myeloblastosis
NAC: NAM–ATAF1,2–CUC2
PR: Pathogen-related
qRT-PCR: Quantitative RT-PCR
SA: Salicylic acid
SAR: System acquired resistance
SCWs: Secondary cell walls
THT: Tyramine hydroxycinnamoyl transferase
WGCNA: Weighted gene co-expression network analysis
WGD: Whole-genome duplication
