# Supplementary material for: Genome-wide identification and expression analysis of the WRKY transcription factor family in flax (Linum usitatissimum L.)
Source: BMC Genomics. 2021 May 22;22:375. doi: 10.1186/s12864-021-07697-w (PMC8141250; doi:10.1186/s12864-021-07697-w)
Supplement: Supplementary file 5 — Additional file 5 Table S5. Primers used for qRT-PCR analysis. [file 12864_2021_7697_MOESM5_ESM.docx]

**Supplemental table S5 Primers used for qRT-PCR analysis**

| Gene ID | Gene Name | Forward primer sequences (5'-3') | Reverse primer sequences (5'-3') |
| --- | --- | --- | --- |
| Lus10007296 | *LuCesA8* | TGAACGACGAATCCGGGAAC | AGTGGCTGTGTTGCATCTCC |
| Lus10007538 | *LuCesA3* | GAAACAGCAGAATTTGCTCG | TGACAAAAGAAGGCTGAACC |
| Lus10008225 | *LuCesA4* | ACTTGTGATTGCTGGCCTTC | AAGTCCTCCAAGTAGGCTTC |
| Lus10012870 | *LuWRKY83* | GAACCCGAGTGAAGTGATG | TCATAGTTACTGATTGCCTCC |
| Lus10013967 | *LuNAC10* | CGAGACGGTCAAAGGGTTC | TTGTTGCCAAGGTGGTATT |
| Lus10020832 | *LuWRKY47* | GAGCCGAGGTTCAGTTTCA | ATGCCTTCCTTCATAGGTTGT |
| Lus10023099 | *LuWRKY86* | GGATTGGAAGGTCCTATTGA | GCTTTATTGCCCAGCAGTTT |
| Lus10039610 | *LuMyb46* | CATCATCAACACCGTCGTCC | TGAATTGATCCCAAGCTGCA |
| CV478202 | *LuGAPDH* | AGGTTCTTCCCGCTCTCAAT | CCTCCTTGATAGCAGCCTTG |
| GR508911 | *LuEF1A* | GCTGCCAACTTCACATCTCA | GATCGCCTGTCAATCTTGGT |
| GR508912 | *LuETIF5A* | TGCCACATGTGAACCGTACT | CTTTACCCTCAGCAAATCCG |
